# A cluster‐randomized controlled trial of a combination HIV risk reduction and economic empowerment intervention for women engaged in sex work in Uganda

**DOI:** 10.1002/jia2.70057

**Published:** 2025-11-10

**Authors:** Susan S. Witte, Fred M. Ssewamala, Joshua Kiyingi, Scarlett L. Bellamy, Lyla Sunyoung Yang, Proscovia Nabunya, Ozge Sensoy Bahar, Larissa Jennings Mayo‐Wilson, Yesim Tozan, Abel Mwebembezi, Joseph Kagaayi

**Affiliations:** ^1^ School of Social Work Columbia University New York New York USA; ^2^ Brown School Washington University in St. Louis St. Louis Missouri USA; ^3^ International Center for Child Health and Development Masaka Field Office Masaka Uganda; ^4^ Division of Prevention Science Department of Medicine University of California San Francisco San Francisco California USA; ^5^ School of Public Health Boston University Boston Massachusetts USA; ^6^ Gillings School of Global Public Health University of North Carolina at Chapel Hill Chapel Hill North Carolina USA; ^7^ School of Global Public Health New York University New York New York USA; ^8^ Reach the Youth Uganda Kampala Uganda; ^9^ Rakai Health Sciences Program Kalisizo Uganda

**Keywords:** economic empowerment, female sex workers, HIV prevention, women engaged in sex work

## Abstract

**Introduction:**

Women engaged in sex work (WESW) in Uganda face a high risk of HIV and other sexually transmitted infections (STIs), driven by the intersection of gender inequality, poverty and structural barriers. This paper reports on the Kyaterekera Project, a cluster‐randomized controlled trial (c‐RCT) testing the efficacy of a combined HIV risk reduction (HIVRR) and economic empowerment intervention to reduce biologically confirmed STIs and HIV risk behaviours.

**Methods:**

The study recruited 542 WESW from 19 HIV hotspots across four districts in Uganda between June 2019 and March 2020. Participants were randomized into three groups: (1) HIVRR intervention alone; (2) HIVRR combined with financial literacy training and an unconditional matched savings account; or (3) HIVRR combined with financial literacy training and an unconditional matched savings account and vocational training. Although initially implemented as a three‐arm c‐RCT, the COVID‐19 lockdown prevented the implementation of the vocational training component. Therefore, the two treatment groups were combined, and the trial was re‐approved as a two‐arm c‐RCT. Biological assessments were conducted at baseline, 18 and 24 months. Behavioural assessments were conducted at baseline, 6, 12, 18 and 24 months from April 2019 to December 2023. Primary outcomes included incident HIV acquisitions (seroconversions among baseline HIV‐negative participants), point prevalence of STIs at each visit, and the number/proportion of unprotected sexual acts with paying and regular partners. This study utilized community‐based participatory research methods, engaging a community advisory board to ensure the study's alignment with local needs.

**Results:**

Across follow‐up, condomless sex with paying partners decreased and income shifted towards non‐sex work in both arms; no between‐group differences were detected. Eighteen incident HIV acquisitions occurred (14 by 18 months; 4 additional by 24 months) with no between‐group differences. STI prevalence was lower at 18 months compared to baseline, but not sustained at 24 months.

**Conclusions:**

In an environment of high baseline HIV prevalence, substantial pre‐exposure prophylaxis uptake and COVID‐19 disruptions, the added financial literacy/savings components did not yield measurable incremental benefits over HIVRR alone. Integrating an unconditional matched‐savings model within an HIVRR platform was feasible.

**Clinical Trial Number:**

NCT03583541

## 1 INTRODUCTION

While the global incidence of human immunodeficiency virus (HIV) is declining, sub‐Saharan Africa continues to bear a disproportionate burden, with HIV prevalence reaching one in every 25 adults [1]. In Uganda, the HIV epidemic persists, with an estimated 1.4 million people living with the virus as of 2021, disproportionately affecting women, who accounted for 57% of new HIV acquisitions [2]. Globally, women engaged in sex work (WESW) are at a significantly higher risk of HIV acquisition compared to the general population [1, 3].

The intersection of gender inequality and poverty exacerbates HIV vulnerability among WESW, necessitating integrated approaches that pair economic empowerment and biomedical interventions. Global consensus emphasizes that HIV epidemic control requires coordinated primary prevention (preventing acquisition) and secondary prevention (reducing onward transmission) delivered through combination prevention that integrates biomedical, behavioural and structural components [4, 5]. Expert reviews also argue for strategies tailored to key populations, including WESW in Uganda [6, 7]. Individual‐level interventions (e.g. condom distribution and HIV education), while necessary, fall short of addressing the structural and economic determinants of HIV risk [6, 7]. Financial insecurity is linked to HIV risk through reduced negotiating power, reliance on sex work income and acceptance of higher‐paying condomless sex [8]. Economic empowerment interventions such as microfinance programmes, cash transfers, vocational training and matched savings enhance women's access to financial resources, opportunities, autonomy and decision‐making power [9, 10, 11].

Few economic empowerment studies target WESW [12]. Recent research shows mixed results regarding the efficacy of combined HIV risk reduction (HIVRR) and economic empowerment interventions. Some show that incorporating an economic component can significantly alter risk behaviours, reducing paying partners and increasing condom use among WESW [13, 14, 15]. A cluster randomized controlled trial in Kazakhstan that evaluated the efficacy of a combination HIVRR and micro‐savings intervention among WESW who use drugs reported no significant differences in HIV and sexually transmitted infection (STI) incidence between groups but noted significant reductions in risky sexual and drug use behaviours, improvements in financial outcomes, attitudes towards condom use, self‐efficacy, social support and reductions in sexual violence from paying partners across both groups [16, 17]. Treatment group participants demonstrated significantly improved financial self‐efficacy compared to those in the control group at 12‐month follow‐up [16]. A recent systematic review of cash transfer programmes for HIV prevention found only one manuscript targeting WESW, reporting that most evidence is limited to demonstrating that cash transfers can reduce HIV acquisition or have long‐lasting impacts on risky sexual behaviours [10].

The Kyaterekera Project study seeks to add to the limited data on how a combination HIVRR and economic strengthening intervention impacts biologically confirmed STIs and HIV risk/incident acquisitions in Uganda. Unlike previous interventions, this study employs an unconditional matched savings account, honouring women's autonomy in financial decision‐making. To our knowledge, it is also the first study to utilize stakeholder engagement involving WESW in Uganda [18]. We hypothesized that a savings‐led economic empowerment intervention group would have (1) a lower incidence of new HIV acquisition; (2) a lower prevalence of biologically confirmed STIs, including gonorrhoea, trichomoniasis and chlamydia; and (3) a lower reported number and proportion of unprotected sexual acts with regular and paying partners.

## 2 METHODS

### 2.1 Recruitment of participants

The Kyaterekera Project is a longitudinal study to test the efficacy of economic components in traditional HIVRR approaches to reduce new cases of HIV and other STIs among WESW in Uganda [19]. The project employed a cluster‐randomized experimental design, recruiting 542 women from 19 HIV hotspots in four districts of Southern Uganda between June 2019 and March 2020 [19]. This study employed community‐based participatory research principles, engaging a community advisory board to guide the study design, recruitment and follow‐up strategies, and ensuring alignment with cultural norms and ethical standards. Community stakeholders contributed to the ethical oversight of the project, ensuring alignment with local priorities [18].

### 2.2 Randomization

**Figure 1:**
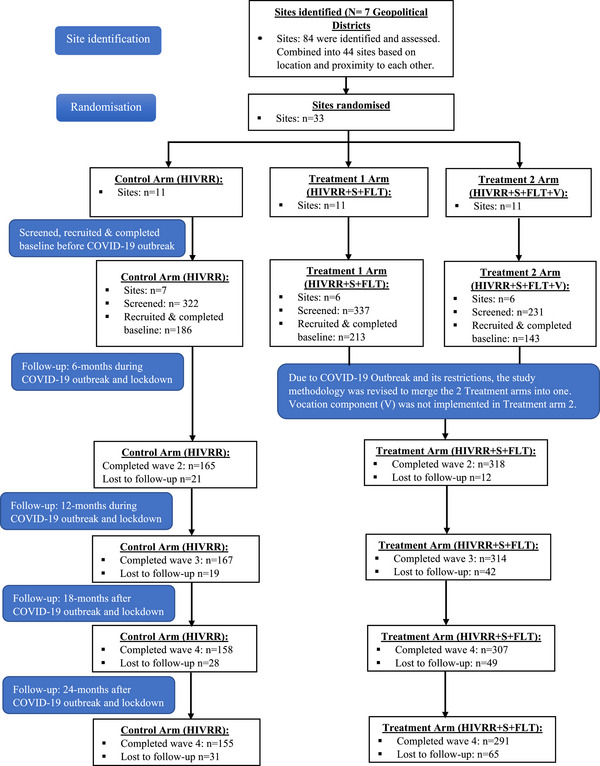
Consort flow diagram.

We used a block randomization approach, with varying block sizes, to allocate 19 geographic hotspot towns to three interventions: HIVRR, HIVRR + S + FL or HIVRR + S + FL + V. Towns were matched into triplets based on their rural or urban status, and the estimated number of WESW to ensure similarity. To avoid contamination, no two hotspots in any triplet were within the same district. After matching, towns were randomized to receive the same intervention. Due to COVID‐19 lockdowns affecting the third treatment group—the addition of vocational training—we combined the two treatment groups (now HIVR+S+FL), and funders reapproved the trial as a two‐group c‐RCT (see Figure 1).

### 2.3 Screening eligibility

Eligibility criteria included: (1) being at least 18 years old; (2) reporting engagement in vaginal or anal sexual intercourse for money, alcohol or other goods in the past 30 days; and (3) reporting at least one episode of unprotected sexual intercourse in the past 30 days with a paying, casual or regular partner. Living with HIV was not an exclusion criterion, as secondary prevention is essential to address the same structural drivers that shape risk for women not living with HIV [4, 5]. In addition to collecting socio‐demographic data, blood and vaginal swab samples were obtained from participants to test for gonorrhoea, chlamydia, trichomonas and HIV. All participants were connected with antiretroviral therapy, STI treatment or pre‐exposure prophylaxis (PrEP) based on need and interest [20]. Local study health collaborators provided testing, counselling and referral to treatment for patients, working with community stakeholders [18].

### 2.4 Intervention

Details on intervention components have been published elsewhere [19].

#### 2.4.1 Control condition (HIVRR)

Participants in the control condition (and treatment group) received four sessions of HIVRR over 2 weeks (see Figure 1), from an evidence‐based HIV/STI risk reduction intervention tested in three previous studies by Witte [15, 16, 21]. These sessions aimed to improve communication, problem‐solving and self‐efficacy for safe sexual practices, with specific training on PrEP and medication adherence during Session 3. The HIVRR session was delivered by community health workers trained and supervised by the study team.

#### 2.4.2 HIVRR+S + FL treatment condition

Women in this group received additional training and support using matched savings accounts and financial literacy (FL) training, emphasizing the importance of savings, banking services, budgeting and debt management. Behavioural economic principles were incorporated to promote safe sexual practices and long‐term financial rewards. Savings accounts were matched at a 1:1 rate monthly, capped at ∼15 USD for 10 months, with equal incentives deposited into personal bank accounts. Participants received monthly bank statements for motivation and could use their savings freely without any restrictions.

#### 2.4.3 HIVRR+S + FL + V treatment condition

While initially designed as a third group, a second treatment group including HIVRR+S+FL, followed by eight vocational skills training, this component of the economic empowerment was shut down due to COVID‐19 restrictions. Mentorship sessions (V) would have supported participants’ transition to vocational, educational or business development, using their matched savings for short‐term and/or long‐term consumption and skills development, at participants’ discretion. This condition was combined with HIVRR+S+FL for data analysis.

### 2.5 Baseline and follow‐up assessments

Trained research assistants collected assessment data from April 2019 to December 2023 at baseline, 6, 12, 18 and 24 months using a 90‐minute questionnaire translated into Luganda by experts from Makerere University to ensure accuracy. Blood samples and vaginal swabs were taken at baseline, 18 and 24 months by medical personnel from Rakai Health Sciences Program, who also provided counselling and referral for any positive test results.

### 2.6 Outcomes

Primary outcomes are aligned with study hypotheses, including:

1. Point prevalence of biologically confirmed STIs (gonorrhoea, chlamydia or trichomoniasis);
2. Incident HIV acquisitions;
3. Reported number and proportion of unprotected vaginal and anal sexual acts with regular and paying partners.

We prespecified several secondary economic outcomes; for this manuscript, we focus on one—proportion of income from sex work—due to its theorized role in linking financial components to sexual risk [22, 23].

### 2.7 Measures

A complete list of measures is published elsewhere [19].

*Socio‐demographics* assessed included age, marital status, education and household size.

*Sexual risk behaviours* were evaluated using the revised Risk Behavior Assessment [24], which assesses the type and number of sexual partners in the past 90 days, as well as sex acts and condom use. If condom use was less than the number of sexual acts, the difference indicated unprotected encounters. The proportion of unprotected sexual acts was calculated by dividing the number of unprotected acts by the total sexual acts for both regular and paying partners.

*Biotesting* focused on three STIs (gonorrhoea, chlamydia and trichomoniasis) given their high prevalence among WESW and the availability of single‐dose treatments. Vaginal swabs were collected for testing: *Neisseria gonorrhoeae* and *Chlamydia trachomatis* were assessed using Nucleic Acid Amplification Tests, while *Trichomonas vaginalis* was tested via culture. NOVA was used for gonorrhoea, Vaxpert for Chlamydia and JD Biotech for Trichomonas. Blood samples for HIV testing and viral load were collected at baseline, 18 and 24 months post‐intervention, employing two enzyme immunoassays for HIV‐1 serostatus confirmation. Abbott Determine, Chembio statpak cassette and Abbott Bioline tests were used for HIV testing. Immediate referrals and treatment were provided for participants testing positive for HIV or STIs. Participants testing positive for HIV were deemed positive at all subsequent follow‐ups. However, other diagnosed STIs were treated immediately, and if STI positive at subsequent assessments, were considered new acquisitions.

*Financial outcomes* were assessed by having participants report their average total income and the proportion of their income from sex work. The proportion of income from sex work was calculated as the average monthly total income as the denominator and income from sex work as the numerator.

### 2.8 Ethics and informed consent

All study procedures were approved by the Washington University in St. Louis Institutional Review Board (IRB #201811106), Columbia University IRB (IRB #AAAR9804) and the local IRBs in Uganda, including the Uganda Virus Research Institute (UVRI #GC/127/18/10/690) and the Uganda National Council of Science and Technology (UNCST #SS4828). Written consent was obtained from participants, with forms translated into Luganda by a certified translator. Each participant received a copy of the consent form.

### 2.9 Statistical analysis

Power analyses were initially designed to detect differences in HIV and STI incidence across three study groups: HIVRR alone, HIVRR + S+FL and HIVRR + S + FL+VT [19]. However, due to the COVID‐19 pandemic and the inability to implement VT, recalculations in August 2020 focused on a simplified primary analysis comparing the combined intervention (HIVRR + S+FL+VT and HIVRR + S + FL) to HIVRR alone. This adjustment eliminated the need for a Bonferroni correction for multiple comparisons. The revised calculations used approximately 180 participants per group and accounted for 80% attrition over a 24‐month follow‐up period.

The revised power calculations indicated that the study would achieve 80% power if STI incidence in the HIVRR alone group was 20–30%, and incidence in the combined intervention groups was 10–18%. We determined that randomizing 180 participants per group (540 total) would provide sufficient power (at least 80%) to detect odds ratios (ORs) of approximately 0.39 or less, representing a clinically significant reduction in incident STIs for participants in the intervention group compared to the HIVRR group. These adjustments ensured that the study remained adequately powered.

We employed statistical methods consistent with the intention‐to‐treat approach. We described the characteristics of study participants at baseline and provided empirical summaries of these characteristics across groups. We presented means and standard deviations for continuous covariates and frequencies and percentages for categorical covariates. We employed a generalized estimating equation (GEE) modelling approach to estimate intervention efficacy. Specifically, we used logistic GEE for the following outcomes: HIV and STI incidence, proportion of unprotected sexual acts, and proportion of income from sex and non‐sex work, and reported estimated ORs and corresponding 95% confidence intervals (CIs). Efficacy for count outcomes (the number of unprotected sexual acts) was estimated using a Poisson GEE approach and reported estimated rate ratios (RRs) and corresponding 95% CIs. Initially, each model contained fixed effects for the study group, time (categorical) and a group‐by‐time interaction. However, we removed the group‐by‐time interaction in the final models when those effects were not significant. We repeated this approach in a sub‐analysis stratified by baseline HIV status. All analyses were conducted in SAS 9.4 [25].

## 3 RESULTS

Results focus on differences between the HIVRR alone group and the combined intervention groups (HIVRR + S+FL). We screened 890 individuals, of whom 542 met eligibility criteria and completed the baseline assessment. Of these, 186 were randomized to the control (HIVRR), and 356 were randomized to the HIVRR+S+FL groups. Intervention groups were homogeneous, with the exception of household size, where the HIVRR+S+FL was 3.8 (SD = 2.4) higher than HIVRR 2.3 (SD = 1.6). Over 87% of the women had primary education, and 25.6% reported being married or in a relationship. At baseline, 41% of participants tested HIV positive, and 10.5% tested positive for at least one STI, including trichomonas (7.4%), chlamydia (2.6%) and gonorrhoea (1.3%). The retention rate at 24 months was 82.3% (see Figure 1). Of participants who were HIV negative at enrolment, 172 (53.4%) accepted PrEP. Table 1 presents baseline characteristics.

**Table 1:** Descriptive statistics of the sample reported at the baseline assessment

	Total (*N* = 542)	HIVRR (*n* = 186)	HIVRR+S+FL (*n* = 356)	*p*‐value
Age (Min/Max: 18–55), mean (SD)	31.4 (7.2)	31.2 (6.8)	31.4 (7.4)	0.8
Education level, *n* (%)				
Primary education	473 (87.7)	167 (89.8)	306 (86.0)	0.2
Secondary school education	69 (12.7)	19 (10.2)	50 (14.0)	
Marital status, *n* (%)				
Married/in a relationship	139 (25.6)	53 (28.5)	86 (24.2)	0.6
Single	72 (13.3)	24 (12.9)	48 (13.5)	
Other (divorced, separated, widowed)	331 (61.1)	109 (58.6)	222 (62.4)	
Household size (Min/Max: 1–18)	3.6 (2.2)	3.26 (1.6)	3.8 (2.4)	0.0
HIV, *n* (%)	220 (41.0)	79 (42.5)	141 (39.6)	0.5
STIs, *n* (%)	57 (10.5)	24 (13.0)	33 (9.3)	0.2

Abbreviations: HIV, human immunodeficiency virus; HIVRR, HIV risk reduction intervention (standard of care); HIVRR+S+FL, combined intervention including HIV risk reduction, savings and financial literacy; *n*, # of participants; STIs, sexually transmitted infections (chlamydia, gonorrhoea, trichomonas).

Table 2 summarizes the distribution of outcomes by group and time point. Table 3 provides study group differences, ORs and corresponding 95% CIs within each time point for study outcomes.

**Table 2:** Summary of study outcomes by study group and time point

		Baseline		6 months		12 Months		18 Months		24 Months	
Outcomes	Study group	*n*		*n*		*n*		*n*		*n*	
STIs: *n* (%)	HIVRR	186	24 (12.9)			NA	NA	145	33 (20.9)	143	17 (11.0)
	HIVRR+S+FL	356	33 (9.3)			NA	NA	286	42 (13.7)	268	39 (13.4)
	Total	542		NA	NA	NA	NA	431		411	
HIV: *n* (%)	HIVRR	186	79 (42.5)	NA	NA	NA	NA	145	67 (42.4)	143	65 (41.9)
	HIVRR+S+FL	356	141 (39.6)	NA	NA	NA	NA	286	123 (40.1)	268	117 (40.2)
	Total	542		NA	NA	NA	NA	431		411	
											
New incident HIV:	HIVRR	186	NA	NA	NA	NA	NA	145	5	143	3
	HIVRR+S+FL	356	NA	NA	NA	NA	NA	286	9	269	1
	Total	542	NA	NA	NA	NA	NA	431	14	411	4
**Number of sexual acts**											
Paying partner^a^: Mean (SD)	HIVRR	186	5.3 (12.0)	165	2.7 (3.4)	167	2.8 (3.0)	158	3.1 (4.5)	155	2.6 (30.5)
	HIVRR+S+FL	356	5.7 (12.0)	318	3.0 (3.4)	314	2.3 (3.1)	307	2.9 (4.0)	291	2.5 (2.8)
	Total	542	5. 6 (11.5)	483	2.9 (3.4)	481	2.7 (3.1)	465	3.0 (4.2)	446	2.5 (2.9)
Regular partner^b^: Mean (SD)	HIVRR	186	4.8 (9.3)	165	4.5 (7.0)	167	4.71(7.13)	158	4.7 (7.8)	155	4.7 (8.6)
	HIVRR+S+FL	356	5.3 (10.0)	318	5.0 (7.8)	314	5.6 (7.8)	307	4.4 (8.0)	291	5.6 (8.3)
	Total	542	5.1 (9.7)	483	4.8 (7.6)	481	5.3 (7.6)	465	4.5 (7.9)	446	5.0 (8.5)
**Number of condoms used**											
Paying partner: Mean (SD)	HIVRR	186	2.3 (3.2)	165	2.3 (3.2)	167	2.5 (3.1)	158	2.2 (3.8)	155	2.1 (3.0)
	HIVRR+S+FL	356	3.5 (7.1)	318	2.5 (2.8)	314	2.3 (2.9)	307	2.0 (3.1)	291	1.9 (2.5)
	Total	542	3.1 (6.1)	483	2.5 (2.9)	481	2.4 (3.0)	465	2.1 (3.4)	446	2.0 (2.7)
Regular partner: Mean (SD)	HIVRR	186	1.2 (5.6)	165	0.8 (2.9)	167	0.7 (1.6)	158	0.4 (1.6)	155	0.4 (2.2)
	HIVRR+S+FL	356	1.2 (4.8)	318	0.8 (2.7)	314	1.1 (3.5)	307	0.4 (1.7)	291	0.3 (1.5)
	Total	542	1.2 (5.1)	483	0.8 (2.7)	481	1.0 (3.0)	465	0.4 (1.7)	446	0.4 (1.8)
**Number of unprotected sex acts**											
Paying partner: Mean (SD)	HIVRR	186	3.1 (11.8)	165	0.5 (1.6)	167	0.4 (1.0)	158	0.9 (2.2)	155	0.5 (1.2)
	HIVRR+S+FL	356	2.4 (8.9)	318	0.5 (2.2)	314	0.4 (1.4)	307	1.0 (3.5)	291	0.6 (1.9)
	Total	542	2.6 (10.0)	483	0.5 (2.0)	481	0.4 (1.3)	465	1.0 (3.1)	446	0.6 (1.7)
Regular partner: Mean (SD)	HIVRR	186	4.0 (9.0)	165	3.7 (6.2)	167	4.1 (7.0)	158	4.2 (7.7)	155	5.2 (8.3)
	HIVRR+S+FL	356	4.5 (9.4)	318	4.2 (7.5)	314	4.5 (7.3)	307	4.0 (7.9)	291	4.4 (8.5)
	Total	542	4.3 (9.3)	483	4.1 (7.1)	481	4.3 (7.2)	465	4.1 (7.8)	446	4.7 (8.4)
**Proportion of unprotected sexual acts** ^c^											
Paying partner: Median (Q25, Q75)	HIVRR	183	0.4 (0, 1)	150	0.1 (0, 0)	143	0.1 (0, 0)	135	0.2 (0, 0.3)	132	0.2 (0, 0)
	HIVRR+S+FL	341	0.4 (0, 0.8)	278	0.1 (0, 0)	276	0.1 (0, 0)	252	0.2 (0, 0.2)	242	0.2 (0, 0)
	Entire sample	524	0.4 (0, 0.9)	428	0.1 (0, 0)	419	0.1 (0, 0)	387	0.2 (0, 0.3)	374	0.2 (0, 0)
Regular partner: Median (Q25, Q75)	HIVRR	103	0.9 (1, 1)	93	0.8 (0.8, 1)	93	0.8 (0.7, 1)	82	0.9 (1, 1)	89	0.87(1, 1)
	HIVRR+S+FL	209	0.8 (1, 1)	179	0.8 (0.8, 1)	204	0.8 (0.7, 1)	167	0.9 (1, 1)	163	0.88(1, 1)
	Total	312	0.8 (1, 1)	272	0.8 (0.8, 1)	297	0.8 (0.6, 1)	249	0.9 (1, 1)	252	0.88(1, 1)
**Proportion of sex work income** ^d^: Median (Q25, Q75)	HIVRR	186	0.9 (0.7, 1)	165	0.7 (0.5, 1)	166	0.6 (0.4, 0.8)	156	0.6 (0.4, 0.8)	155	0.6 (0.4, 0.8)
	HIVRR+S+FL	356	0.9 (0.8, 1)	318	0.7 (0.5, 1)	312	0.7 (0.4, 1)	305	0.6 (0.3, 0.8)	285	0.6 (0.3, 1)
	Total	542	0.9 (0.8, 1)	483	0.7 (0.5, 1)	478	0.6 (0.4, 0.9)	461	0.6 (0.3, 0.8)	440	0.6 (0.4, 0.9)
**Proportion of non‐sex work income**: Median (Q25, Q75)	HIVRR	186	0.2 (0, 0.3)	165	0.3 (0, 0.5)	166	0.4 (0.2, 0.6)	156	0.4 (0.2, 0.6)	155	0.4 (0.2, 0.6)
	HIVRR+S+FL	356	0.1 (0, 0.2)	318	0.3 (0, 0.5)	312	0.4 (0.0, 0.6)	305	0.5 (0.2, 0.7)	285	0.4 (0, 0.7)
	Total	542	0.1 (0, 0.3)	483	0.3 (0, 0.5)	478	0.4 (0.1, 0.6)	461	0.4 (0.2, 0.7)	440	0.4 (0.1, 0.6)

Abbreviations: HIV, human immunodeficiency virus; HIVRR, HIV risk reduction intervention (standard of care); HIVRR+S+FL, combined intervention including HIV risk reduction, savings and financial literacy components; *n*, # of participants; NA, not applicable; OR, odds ratio; Q25, Q75, 25th and 75th percentiles (interquartile range); RR, rate ratio; SD, standard deviation; STIs, sexually transmitted infections (chlamydia, gonorrhoea, trichomonas).

^a^ “Paying partner” refers to a transactional partner.

^b^“Regular partner” refers to a stable or non‐transactional partner.

^c^“Proportion of unprotected sexual acts” refers to unprotected acts divided by total acts with each partner type.

^d^“Proportion of income from sex work” and “Proportion of non‐sex work income” reflect the respective income source as a share of overall income.

**Table 3:** Study group differences and odds ratios within each time point for the study outcomes

	STIs	HIV	Number of unprotected sexual acts (paying partner)^a^	Number of unprotected sexual acts (regular partner)^b^	Proportion of unprotected sexual acts^c^ (paying partner)	Proportion of unprotected sexual acts (regular partner)	Proportion of total income from sex work^d^	Proportion of total income not from sex work
	**OR (95%CI)**	**OR (95%CI)**	**RR (95%CI)**	**RR (95%CI)**	**OR (95%CI)**	**OR (95%CI)**	**OR (95%CI)**	**OR (95%CI)**
**HIVRR (reference group)**								
HIVRR+S+FL	1.3 (0.9, 1.9)	1.1 (0.8, 1.6)	0.9 (0.6, 1.4)	1.0 (0.8, 1.3)	0.8 (0.5, 1.3)	1.0 (0.7, 1.5)	1.2 (1.0, 1.3)	0.9 (0.8, 1.0)
**Baseline (reference group)**								
6 months	‐		0.2 (0.1, 0.3)	0.9 (0.8, 1.1)	0.2 (0.1, 0.4)	1.0 (0.6, 1.6)	0.4 (0.3, 0.5)	2.5 (2.0, 3.0)
12 months	‐		0.1 (0.1, 0.2)	1.0 (0.8, 1.2)	0.2 (0.1 0.3)	0.9 (0.6, 1.4)	0.3 (0.3, 0.4)	2.9 (2.4, 3.6)
18 months	0.6 (0.4, 0.8)	0.9 (0.8, 1.0)	0.4 (0.2, 0.6)	0.9 (0.7, 1.2)	0.5 (0.3, 0.9)	1.8 (1.0, 3.1)	0.3 (0.3, 0.4)	3.1 (2.5, 3.9)
24 months	0.8 (0.5, 1.1)	0.9 (0.8, 1.0)	0.2 (0.1, 0.3)	1.1 (0.9, 1.4)	0.3 (0.2, 0.5)	2.4 (1.3, 4.3)	0.4 (0.3, 0.4)	2.8 (2.3, 3.5)

Abbreviations: HIV, human immunodeficiency virus; HIVRR, HIV risk reduction intervention (standard of care); HIVRR+S+FL, combined intervention including HIV risk reduction, savings and financial literacy; *n*, number of participants; OR, odds ratio; RR, rate ratio; STIs, sexually transmitted infections (chlamydia, gonorrhoea, trichomonas).

^a^ “Paying partner” refers to a transactional partner.

^b^“Regular partner” refers to a stable or non‐transactional partner.

^c^“Proportion of unprotected sexual acts” refers to unprotected acts divided by total acts with each partner type.

^d^“Proportion of income from sex work” and “Proportion of non‐sex work income” reflect the respective income source as a share of overall income.

*Incident HIV acquisitions* were observed among participants over 24 months. At 18 months, there were 14 cases (five in the HIVRR and nine in the HIVRR+S+FL group), and an additional four cases were recorded at 24 months (three in HIVRR and one in HIVRR+S+FL). However, there were no significant differences between the groups.

*STI prevalence* fluctuated over time. In the HIVRR group, prevalence increased from 12.9% at baseline to 20.9% at 18 months before declining to 11% by 24 months. In the HIVRR+S+FL group, STI prevalence increased from 9.3% at baseline to 13.7% at 18 months and remained stable at 13.4% at 24 months. Modelled outcomes in Table 3 indicate significantly lower odds of STI at 18 months compared to baseline in both groups (OR = 0.6, 95% CI = 0.4−0.8). However, no significant differences were observed between groups at 18 or 24 months.

*Sexual risk behaviour* with paying partners decreased significantly in both groups. In the HIVRR group, the median proportion of unprotected acts decreased from 0.4 at baseline to 0.1 at 6 months, staying below 0.2 throughout the study. The HIVRR+S+FL group decreased from 0.4 at baseline to 0.1 by 6 months, with a slight increase to 0.2 by 24 months. At 24 months, Table 3 shows significantly lower odds of condomless sex with paying partners (OR = 0.3, 95% CI = 0.2−0.5, *p*<0.0). Conversely, unprotected sex with regular partners showed less consistent changes, returning to baseline or higher by 18 and 24 months, with significantly higher odds of condomless sex at 24 months compared to baseline (OR = 2.4, 95% CI = 1.3−4.3, respectively), with no significant differences between groups.

*Economic outcomes* improved, with the proportion of income from sex work decreasing from 0.9 to 0.6 in HIVRR and from 0.9 to 0.6 in HIVRR+S+FL at 24 months. Non‐sex work income significantly increased in both groups. Table 3 shows notable reductions in income from sex work at 24 months (OR = 0.45, 95% CI = 0.3−0.4, *p*< 0.0), while median non‐sex work income rose. No significant group differences were found. Reported non‐sex work income sectors included hospitality (e.g. bar/restaurant), sales/small trade 15.8%, personal care 6.3% (e.g. salon/beauty), tailoring/craft 5.7% (e.g. sewing/painting) and community 5.1% (peer educator, NGO outreach/supervision).

*Stratified analyses by HIV status* (Tables 4 and 5) reveal that among HIV‐negative participants, both groups showed improved outcomes over time, such as reduced unprotected sex with paying partners and increased non‐sex work income, with no significant differences between groups. Among participants living with HIV, while unprotected sex acts and income patterns were similar, STI incidence was significantly higher in the HIVRR+S+FL group compared to HIVRR (OR = 2.1, 95% CI = 1.3−3.5, *p* < 0.0), with no other significant differences noted.

**Table 4:** Study group differences and odds ratios within each time point for the study outcomes—HIV‐negative participants

	STIs	Number of unprotected sexual acts (paying partner)w^a^	Number of unprotected sexual acts (regular partner)^b^	Proportion of unprotected sexual acts^c^ (paying partner)	Proportion of unprotected sexual acts (regular partner)	Proportion of total income from sex work^d^	Proportion of total income not from sex work
	**OR (95%CI)**	**RR (95%CI)**	**RR (95%CI)**	**OR (95%CI)**	**OR (95%CI)**	**OR (95%CI)**	**OR (95%CI)**
**HIVRR (reference group)**							
HIVRR+S+FL	**0.8 (0.4, 1.3**)	1.1 (0.7, 1.6)	1.2 (0.9, 1.5)	1.0 (0.6, 1.5)	01.0 (0.6, 1.6)	1.1 (1.0, 1.3)	**0.9 (0.8, 1.1)**
**Baseline (reference group)**							
6 months	‐	0.2 (0.1, 0.5)	1.0 (0.8, 1.2)	0.3 (0.1, 0.5)	0.93 (0.52, 1.64)	0.5 (0.4, 0.6)	2.2 (1.7, 2.9)
12 months	‐	0.2 (0.1, 0.4)	1.2 (0.9, 1.5)	0.2 (0.1, 0.4)	1.01 (0.57, 1.77)	0.4 (0.3, 0.5)	2.8 (2.2, 3.7)
18 months	0.5 (0.3, 0.8)	0.5 (0.3, 0.8)	1.0 (0.7, 1.3)	0.7 (0.4, 1.3)	2.02 (1.01, 4.04)	0.3 (0.3, 0.5)	2.9 (2.2, 3.9)
24 months	0.6 (0.4, 1.0)	0.2 (0.1, 0.3)	1.2 (0.9, 1.5)	0.4 (0.2, 0.6)	2.82 (1.35, 5.91)	0.4 (0.3, 0.5)	2.5 (1.9, 3.2)

Abbreviations: HIV, human immunodeficiency virus; HIVRR, HIV risk reduction intervention (standard of care); HIVRR+S+FL, combined intervention including HIV risk reduction, savings and financial literacy; OR, odds ratio; RR, rate ratio; STIs, sexually transmitted infections (chlamydia, gonorrhoea, trichomonas).

^a^ “Paying partner” refers to a transactional partner.

^b^“Regular partner” refers to a stable or non‐transactional partner.

^c^“Proportion of unprotected sexual acts” refers to unprotected acts divided by total acts with each partner type.

^d^“Proportion of income from sex work” and “Proportion of non‐sex work income” reflect the respective income source as a share of overall income.

**Table 5:** Study group differences and odds ratios within each time point for the study outcomes—HIV‐positive participants

	STIs	Number of unprotected sexual acts (paying partner)^a^	Number of unprotected sexual acts (regular partner)^b^	Proportion of unprotected sexual acts^c^ (paying partner)	Proportion of unprotected sexual acts (regular partner)	Proportion of total income from sex work^d^	Proportion of total income not from sex work
	**OR (95%CI)**	**RR (95%CI)**	**RR (95%CI)**	**OR (95%CI)**	**OR (95%CI)**	**OR (95%CI)**	**OR (95%CI)**
**HIVRR (reference group)**							
HIVRR+S+FL	**2.1 (1.3, 3.5)**	0.8 (0.4, 1.7)	0.8 (0.6, 1.2)	0.7 (0.4, 1.4)	1.1 (0.5, 2.4)	1.2 (1.0, 1.5)	**0.8 (0.7, 1.0)**
**Baseline (reference group)**							
6 months	‐	0.2 (0.1, 0.4)	0.9 (0.6, 1.3)	0.2 (0.1, 0.4)	1.2 (0.5, 2.7)	0.3 (0.3, 0.4)	3.0 (2.3, 3.9)
12 months	‐	0.1 (0.1, 0.2)	0.7 (0.5, 1.1)	0.1 (0.1, 0.3)	0.7 (0.3, 1.4)	0.4 (0.3, 0.5)	2.9 (2.2, 3.9)
18 months	0.7 (0.2, 0.4)	0.3 (0.1, 0.5)	0.9 (0.6, 1.4)	0.3 (0.2, 0.7)	1.4 (0.6, 3.3)	0.3 (0.2, 0.4)	3.4 (2.6, 4.5)
24 months	0.9 (0.6, 1.6)	0.2 (0.1, 0.4)	1.0 (0.7, 1.4)	0.4 (0.1, 0.6)	1.8 (0.6, 4.9)	0.3 (0.2, 0.4)	3.5 (2.7, 4.7)

Abbreviations: HIV, human immunodeficiency virus; HIVRR, HIV risk reduction intervention (standard of care); HIVRR+S+FL, combined intervention including HIV risk reduction, savings and financial literacy; OR, odds ratio; RR, rate ratio; STIs, sexually transmitted infections (chlamydia, gonorrhoea, trichomonas).

^a^ “Paying partner” refers to a transactional partner.

^b^ “Regular partner” refers to a stable or non‐transactional partner.

^c^ “Proportion of unprotected sexual acts” refers to unprotected acts divided by total acts with each partner type.

^d^ “Proportion of income from sex work” and “Proportion of non‐sex work income” reflect the respective income source as a share of overall income.

## 4 DISCUSSION

Study results suggest that participants in both the HIVRR control and HIVRR+S+FL treatment groups experienced reductions in sexual risk behaviours and modest improvements in economic outcomes over 24 months. Both groups achieved reductions in the count and proportion of unprotected sex acts with paying partners and increased income from non‐sex work activities. We did not detect incremental effects attributable to the added economic components, and neither group demonstrated significant reductions in HIV or STI prevalence, consistent with evidence that behavioural changes may not always immediately translate into biological outcomes [16, 26]. Substantial PrEP uptake among participants not living with HIV at baseline, an active control (HIVRR in both groups), and few seroconversions (*n* = 18) likely attenuated differences in HIV outcomes between groups and limited the ability to detect differences.

STI prevalence decreased over time in both groups, with the HIVRR+S+FL group showing slightly lower prevalence at 18 months compared to the HIVRR group. The numerically lower 18‐month value in HIVRR+S+FL was non‐significant and not sustained, so it should be interpreted cautiously and does not indicate an incremental effect. Despite reductions in condomless sex with paying partners, persistently high condomless sex with regular partners may have attenuated gains in STI outcomes. This highlights the need for tailored strategies addressing relationship dynamics and barriers to consistent condom use in intimate partnerships [17].

Both groups demonstrated significant reductions in reliance on sex work income and increased non‐sex work income reflecting economic diversification; however, no significant differences in economic outcomes were observed between them. The lack of measurable differences suggests that HIVRR alone may have been sufficient to catalyse economic shifts in this context. These findings raise important questions about the added value of economic components when paired with strong behavioural interventions and the need for further exploration [10, 16]. Transitions mainly shifted into hospitality, service and small trade: informal, low‐wage sectors that align with pandemic restrictions. While diversification may have reduced dependence on sex work, it remained unstable, which could explain the parallel economic trends across groups and the absence of distinct behavioural or biological effects.

The absence of significant differences between groups may reflect the strength of the HIVRR intervention provided to all participants, potentially creating a “threshold effect, ” where changes in both groups limited the ability to detect additional benefit from the economic empowerment components [23]. Ethical guidelines from UNAIDS and WHO mandate inclusion of standard HIV prevention in all study groups, ensuring access to care but limiting the feasibility of placebo controls [20].

Stratified analyses by HIV status revealed that HIV‐negative participants in both groups had substantial reductions in HIV and STI risk behaviours and economic indicators over time, indicating the HIVRR's effectiveness in this subgroup. Among participants living with HIV, outcomes were comparable across groups, except for higher STI incidence in the HIVRR+S+FL group, which may reflect chance variation or reporting differences. No significant differences in unprotected sex or income patterns were observed, underscoring cautious interpretation of the STI finding. These findings reinforce the challenges of achieving added benefit over an active control, and the importance of tailoring interventions to address the distinct prevention and economic needs of participants living with and without HIV.

Finally, the study design highlights the challenge of evaluating new components in the context of strong behavioural interventions. Although not an “active control” trial, this study employed a rigorous HIVRR intervention in both groups. While ensuring ethical rigour, this approach limits the ability to isolate the incremental effects of new components. Future studies could consider using external cohort data or exposure markers to estimate counterfactual outcomes [27, 28, 29].

## 5 LIMITATIONS

The study faced several limitations, including the disruption of vocational training due to the COVID‐19 pandemic, which may have attenuated the intervention's overall impact. Reliance on self‐reported data for sexual behaviour may have introduced response biases. The pandemic significantly disrupted study implementation in Uganda, exacerbating socio‐economic challenges for participants, such as government curfews, restricted movement [30, 31], income loss, food insecurity and housing instability [32, 33], disproportionately affecting WESW who were often excluded from emergency support [34]. Additionally, disruptions in sexual and reproductive health services [35, 36] may have hindered participants’ ability to benefit from the intervention and may have contributed to the 18‐month STI spike and accelerated transitions away from sex work. Future studies should employ more robust measures of both behavioural and economic outcomes and ensure adaptive implementation in the face of historical disruptions.

## 6 CONCLUSIONS

The Kyaterekera Project contributes valuable evidence at the intersection of economic empowerment and HIV prevention among WESW. In a context of high baseline HIV prevalence, substantial PrEP uptake and COVID‐19 disruptions, integrating financial components (including an unconditional matched savings) within a robust HIVRR platform was feasible. However, the added FL and savings components did not yield measurable incremental benefits over HIVRR alone on behavioural or biological outcomes. The design, ensuring all participants receive evidence‐based HIVRR, aligns with ethical standards but complicates the detection of added effects when improvements occur in both groups.

Future research should identify when and for whom economic components confer incremental benefit (e.g. intervention intensity and timing, and structural supports), employ designs powered to detect differences in biological endpoints and consider complementary methods (e.g. external cohort or exposure marker approaches) to isolate component effects. Emphasizing scalability, community engagement, cultural alignment and participant autonomy can enhance intervention acceptability and support sustained improvements in the lives of WESW.

## COMPETING INTERESTS

The authors declare that they have no competing interests.

## AUTHOR CONTRIBUTIONS

SSW and FMS are joint principal investigators who conceptualized the study. SSW and LSY developed a manuscript draft, SLB advised on the analysis, JK conducted the data analysis, and FMS, PN and OSB reviewed the drafts. All authors, including LJM‐W, YT, AM and JK, were instrumental in the implementation of the clinical trial. All authors approved the manuscript submission.

## FUNDING

This study was funded by the National Institute of Mental Health for Ssewamala and Witte (R01MH116768). The funder was not involved in the study design or analysis.

## Data Availability

The research team will make datasets available to any individual who makes a direct request to the PIs and indicates the data will be used for research (per CFR Title 45 Part 46: “Research is defined as a systematic investigation, including research development, testing, and evaluation, designed to develop or contribute to generalizable knowledge”). When sharing participant data, the team will adhere to the data‐sharing agreements established by the Brown School of Social Work and the Columbia University School of Social Work Office of Sponsored Projects.
